# Chronic neurological diseases and COVID-19: Associations and considerations

**DOI:** 10.1515/tnsci-2020-0141

**Published:** 2020-09-09

**Authors:** Shakila Meshkat, Amir Salimi, Asef Joshaghanian, Sogol Sedighi, Saman Sedighi, Vajiheh Aghamollaii

**Affiliations:** Department of Medicine, Tehran University of Medical Sciences, Tehran, Iran; Department of Medicine, Shiraz University of Medical Sciences, Tehran, Iran; Department of Medicine, Shahid Beheshti University of Medical Sciences, Tehran, Iran; Department of Medicine, Azad University of Medical Sciences, Tehran, Iran; Department of Medicine, Hamedan University of Medical Sciences, Tehran, Iran

**Keywords:** COVID-19, coronavirus, epilepsy, Parkinson’s disease, dementia, multiple sclerosis, SARS-CoV-2, chronic neurological diseases, telemedicine

## Abstract

The 2019 novel coronavirus pandemic, severe acute respiratory syndrome CoV-2 (COVID-19), has been a worldwide urgent public health threat, resulting in six-hundred seventy thousand deaths to date. The COVID-19 pandemic has led to a series of public health challenges. One such challenge is the management of diseases such as chronic neurological diseases during an epidemic event. COVID-19 affects all kinds of people, including older people with chronic underlying diseases, who are particularly at risk of severe infection or even death. Chronic neurological diseases such as epilepsy, dementia, Parkinson’s disease (PD), and multiple sclerosis (MS) are frequently associated with comorbidities; thus, these patients are in the high-risk category. Therefore, in this article, we review associations and challenges the people with epilepsy, dementia, PD, and MS faces during the COVID-19 pandemic and suggest approaches to provide consensus recommendations on how to provide the best possible care.

## 1 Introduction

Coronaviruses (CoVs) are enveloped, positive-sense single-stranded viruses ((+)ssRNA virus) belonging to the family Coronaviridae of the Orthocoronavirinae subfamily. Even though most pathogenic subtypes of CoVs in humans are associated with mild clinical features, two notable examples are severe acute respiratory syndrome-related coronavirus (SARS-CoV) and Middle East respiratory syndrome coronavirus (MERS-CoV) [1]. In Saudi Arabia, MERS-CoV was first reported in 2012, which caused 858 deaths. In 2002, a Beta-CoV subtype spread rapidly across Guangdong, China. This outbreak resulted in 8,000 infections and 774 deaths in 37 countries [2].

The Coronavirus disease 2019 (COVID-19) pandemic, which is a novel infectious disease caused by severe acute respiratory syndrome coronavirus two (SARS-CoV-2), was first reported in Wuhan, China [2]. Symptoms of COVID-19 appear after 5 days of the incubation period. However, the incubation period ranges from 6 to 41 days, depending on the patients’ age and immune system status [3]. COVID-19 presents with a wide clinical spectrum from asymptomatic patients to septic shock and multi-organ failure, and it is categorized as mild, moderate, and severe based on presentation severity. Fever, cough, and fatigue are the most common symptoms of COVID-19; other symptoms include headache, hemoptysis, and dyspnea. In addition to the mentioned symptoms, COVID-19 is also associated with neurological manifestations such as febrile seizures, convulsions, change in mental status, and encephalitis [3,4].

Like all respiratory viral agents, transmission routes of SARS-CoV-2 into the central nervous system (CNS) include two major pathways: hematogenous and neuronal retrograde routes [5]. The virus undergoes retrograde axonal transport through the retrograde neuronal pathway to enter the neurons’ cell bodies of the peripheral nervous system and CNS. In the hematogenous route, the virus infects the endothelial cells of the blood–brain barrier and epithelial cells of the blood–cerebrospinal cells in the choroid plexus [6]. Under other conditions, SARS-CoV-2 may use the olfactory nerve to access the brain through the olfactory bulb. Besides, viruses can use other peripheral nerves such as the trigeminal nerve that has nociceptive neural cells in the nasal cavity or the sensory fibers of the vagus nerve that originates from brain stem and innervates multiple respiratory tract organs including the larynx, trachea, and lungs [7]. The CoV can also spread to the brainstem directly through mechanoreceptors and chemoreceptors located in the lung and the lower respiratory tract [8]. Like other well-recognized neuroinvasive human viruses, SARS-CoV-2 can damage the CNS due to misdirected host immune responses that could be associated with autoimmunity in susceptible individuals (virus-induced neuro-immunopathology) [5]. SARS-CoV-2 infection is associated with cytokine dysregulation known as “cytokine storm.” Cytokine storm is defined as the release of cytokines such as interleukin receptor-2, interleukin-6, tumor necrosis factor α, and IL-10 from infected neurons in order to control the infection that can result in neuronal damage, especially in those with low immunity to the virus [9]. In addition to the mentioned pathways, angiotensin-converting enzyme 2 (ACE2) is one of the main receptors that mediate the entry of SARS-CoV-2 into human cells [10]. The virus binds to host cells ACE2 through protein S and infects the cells. ACE2 are predominantly expressed in glial cells (astrocytes, oligodendrocytes, and microglial cells) and neurons mainly located (both excitatory and inhibitory neurons) in human middle temporal gyrus and posterior cingulate cortex [9,10]; consequently, CNS is a highly possible target for SARS-CoV-2 infection.

COVID-19 affects slightly more men with a median age of 56 years. In particular, patients requiring intensive care are older and have various comorbidities such as neurological, cerebrovascular, endocrine, metabolic, and respiratory diseases [11]. Given the global spread and high mortality rate of COVID-19, it is essential to determine possible risk factors affecting the disease progression. Previous studies showed that patients with comorbidities could give rise to poor prognosis. Hence, detecting risk groups is essential when making anti-SARS-CoV-2 therapy decisions [12].

Chronic neurological diseases (epilepsy, dementia, Parkinson’s disease [PD], multiple sclerosis [MS]) and COVID-19 association have not been described enough. In this study, by reviewing known facts of COVID-19 and recommendations from medical societies [13,14,15,16], we discuss the considerations and possible associations in these patients.

## 2 Epilepsy

Epilepsy is one of the most common chronic neurological conditions characterized by the spontaneous recurrence of unprovoked seizures. The prevalence is estimated at 0.7–1.0% with high incidences in the elderly and childhood [17]. Given the absence of data, the Centre for Disease Control and Prevention believes that neurological comorbidities, including seizures, could be a risk factor for COVID-19. However, information from countries with pandemic experience does not suggest epilepsy as a risk factor for developing COVID-19. Epilepsy patients with diseases restricting mobility, respiratory conditions (including asthma), diabetes mellitus, hypertension, severe heart disease, immunodeficiency due to underlying conditions or drug treatment, and older age are at higher risk of severe SARS-CoV-2 infection [18]. Besides, patients with autoimmune disorders that are related to epilepsy and patients with tuberous sclerosis complex (TSC), which is frequently followed by epilepsy, may be at higher risk of COVID-19 due to immunosuppressive agents and reduced lung function. There may also be a risk of worsening in a person with epilepsy where seizures are caused by fever or some epilepsy syndromes such as Dravet syndrome [19,20]. Epilepsy patients with these comorbidities should take a more cautionary approach to COVID-19. Ultimately, children with well-controlled epilepsy who get infected by COVID-19 are usually asymptomatic or have mild symptoms [21].

As we mentioned earlier, CNS is a potential target of SARS-CoV-2. CNS infection can result in seizures, while the presence of SARS-CoV-2 in cerebrospinal fluid (CSF) is uncommon; some case reports have detected SARS-CoV-2 in CSF [22]. COVID-19 can cause variable rates of seizures associated with fever or seizure exacerbation, especially in late/severe stages [9]. Meningitis/encephalitis associated with SARS-CoV-2 followed by seizures is also reported. Another study identified a patient suffering from COVID-19, whose primary presentation was an epileptic focal status epilepticus [23]. However, tubercular meningitis co-occurrence was reported without seizure or status epilepticus. Hypoxia, multi-organ dysfunction, and metabolic and electrolyte derangements may be present in patients with severe COVID-19 and may require complex drug regimens and therapeutic interventions. Thus, it is also realistic to expect these patients to suffer severe or subclinical acute symptomatic seizures and status epilepticus [24]. Further follow-up is required to determine whether COVID-19 can cause direct CNS attacks.

Although there is no specific drug that should be avoided in epileptic patients, scientists do not suggest nonsteroidal anti-inflammatory drugs in the acute phase of SARS-CoV-2 infection. Therefore, in epilepsy patients that fever control is essential (such as Dravet syndrome), acetaminophen (paracetamol) can be used, followed by ibuprofen as needed [25]. Additionally, patients with epilepsy should not use decongestant pseudoephedrine or antihistamine diphenhydramine because they may worsen seizures. To date, there are no approved coronavirus treatments at this time. Several different drugs are being studied as potential COVID-19 therapies to minimize viral load or disease severity. While some of these medications may be effective, they all have adverse effects, and some have possible drug interactions. Some of the adverse effects can include seizures. The drug that’s furthest along in clinical trials for treating COVID-19 is Remdesivir, a new intravenous antiviral that the food and drug administration (FDA) has not yet approved and has no known interaction with anti-epileptic drugs (AEDs) [26,27]. Other tested drugs, including Ribavirin, Nitazoxinide, and Tocilizumab, do not interact with AEDs either [26]. Interaction between COVID-19 treatments and AEDs requires consideration. The AEDs have some interaction with other medicines through drug-metabolizing enzymes and pharmacodynamics interactions [27]. Some AEDs may target the immune system, such as Everolimus and steroids used to treat TSC and autoimmune epilepsy, respectively. Modifying the AEDs of patients with well-controlled seizures because of seizure exacerbations or status epilepticus may raise the risk of COVID-19. In clinical settings, therefore, medications must be chosen on an individual basis [28].

Patients with epilepsy must prevent running out of AEDs. Seizure attacks can cause malnutrition, which is linked to the immune system. Also, the mortality rate is higher in patients with uncontrollable seizures compared with those with controllable seizures [19]. Patients with epilepsy should avoid going to emergency rooms because they can be exposed to the coronavirus. Hence, it is essential to maintain control of seizure as the prevention of COVID-19. However, patients in status epilepticus should still seek full medical attention, including referral to emergency departments for life/brain saving interventions. The hospital and staff must follow health safety protocols to prevent these patients from becoming infected. Elective surgical treatment for epilepsy should be postponed for at least three months to avoid spreading viruses between patients and medical staff. Hospitals should be ready for COVID-19 patients who need critical care. Patients with epilepsy who require critical care should adhere to prevention and protection protocols against COVID-19. Also, those suspected to be infected with SARS-CoV-2 should be isolated.

## 3 Dementia

Dementia is a chronic neurodegenerative disease characterized by a progressive decline of cognitive performance, which has a harmful impact on social activities. Alzheimer’s disease (AD) is the main cause of dementia and loss of functional independence in the elderly. The prevalence is rapidly increasing due to the growth of the proportion of people aged 65 years and older [29].

The risk of COVID-19 may increase in AD patients for several reasons; it is hard for them to follow the recommendation from public health authorities to reduce the risk of infection, such as social distancing, social isolation, covering mouth, nose, wearing masks, etc. Patients with severe dementia due to cognitive impairment and memory loss are unable to understand suggestions. However, severe AD patients who would not be able to follow suggestions would have such a poor quality of life that they would require 24 h of support from caregivers. Communicative and socially restrictive conditions in the COVID-19 pandemic lead to cognitive decline and worsening of the disease stage and increase the risk of acute cognitive changes and delirium [30]. Besides, a very important aspect of care in the elderly with chronic neurologic conditions such as dementia and PD is that many of these people are residents of nursing homes and long-term care facilities. We know from epidemiologic evidence that COVID-19, unfortunately, affects many people in places such as nursing homes, retirement homes, and long-term care facilities. Patients with AD are more likely to have comorbidities such as cardiovascular disease, diabetes, which makes them susceptible to infection [30]. However, AD patients with ApoE e4e4 allele are at higher risk of severe COVID-19 independent of preexisting dementia, cardiovascular disease, and type-2 diabetes [31]. In addition to comorbidities, age is a major risk factor for COVID-19, and most AD patients are older than 65 years [32].

COVID-19 in AD patients may present with atypical symptoms, i.e., namely diarrhea or drowsiness, which can be challenging for physicians to diagnose the disease. Also, a nasopharyngeal swab can be negative and does not exclude COVID-19 if there is a high clinical suspicion. Patients may die from worsening of general health instead of the infection itself. In-hospital complications, including getting bedridden, poor nutrition, and dehydration, are higher in AD patients, possibly leading to more clinical complications and increased mortality rate. Also, dementia can get worsen during infection due to hypoxia, a prominent clinical feature of COVID-19 [30,33].

In patients with AD, the use of anticholinesterase inhibitors and Memantine is common. No potential interaction has been found between Remdesivir, Tocilizumab, Ribavirin, Favipiravir, and AD treatments [34]. Drug reactions in AD patients should be assessed when undergoing treatment with COVID-19. Mainly, better therapies for COVID-19 and AD should be chosen, but the physician should carefully supervise important factors. In addition, vitamin D, which affects the pathogenesis of this disease, should be taken in these patients because low vitamin D can susceptible patients to severe forms of COVID-19 [35,36].

Routine screening such as in-person clinical assessments, blood tests, electrocardiograms, or the ability to follow up on adverse events is disrupted. Thus, changing maintenance drugs or adding new medication is not recommended during the pandemic. Patients who are stable on medications may be affected if their medication’s supply is interrupted due to missed visits, pharmacy collection, or distribution failure, or issues with the supply chain. Hence, drugs should be kept at home.

## 4 PD

PD is the second most common neurodegenerative disorder after AD, with a prevalence of 0.3% in the general population. PD is a brain disorder that is highly associated with age. PD affects 1.0% in people older than 60 years, and 3.0% in those aged 80 years and older. Clinical presentations include motor symptoms such as bradykinesia, resting tremor, rigidity, and postural instability [37].

There is no evidence that PD patients are at higher risk of COVIID-19. However, in a large population study on people older than 55 years, PD patients had more comorbidities such as coronary artery disease, cerebrovascular disease, and heart failure, which are known to render patients at greater risk for severe forms of COVID-19 [38]. Also, age is an established risk factor for a severe form of COVID-19 [32], and PD is a disease that is common in the elderly. Thus, it is logical that people with PD be at higher risk of developing severe SARS-CoV-2 infection. However, the loss of neurons in the nigra-striatal system leads to fewer neurons present for ACE2 binding, and further CNS viral infiltration can protect patients from increased infection [37,39].

The severe form of COVID-19 is more likely to occur in some subgroup patients of PD, those suffering from respiratory dysfunction. Advanced PD patients may have restricted pulmonary capacity due to axial akinesia, which can cause a higher risk for pulmonary decompensation [40]. Besides, PD can involve brainstem nuclei and affect the medulla respiratory center, which is targeted by COVID-19 [9,37]. Consequently, if infected with SARS-CoV-2, PD patients with underlying respiratory dysfunction may be at risk of respiratory failure [39,40]. It is well known that Parkinsonism tends to decompensate with acute stress and particularly with fever, both critical symptoms of COVID-19 [2,39]. Under these circumstances, PD patients are at risk of having extremely generalized akinesia or akinetic crisis, and dopaminergic drugs may need a rapid rise [41].

SARS-CoV-2 enters and affects the brain through the ACE2 receptor, which is highly expressed in dopamine neurons, and they are significantly reduced in PD due to the degenerative process; thus, COVID-19 can cause additional harm and worsen symptoms and may increase the requirement of dopamine replacement therapy [39,42]. The nasal cavity is another site of SARS CoV2 penetration into CNS that determines anosmia/hyposmia and ageusia in many affected individuals, which are common non-motor signs of PD and may lead to delayed diagnosis in these patients [9]. As a result, the dopamine synthetic pathway may be involved in the pathophysiology of COVID-19 as ACE2 and DOPA decarboxylase co-express and co-regulate in non-neuronal cell types that may indicate dopamine depletion and need to be treated with levodopa [43].

Antonini et al., reported 10 PD patients with COVID-19. Nonmotor symptoms such as anxiety, fatigue, orthostatic hypotension, cognitive impairment, and psychosis got worsen during the disease. Older PD patients with longer disease duration had a higher mortality rate and were susceptible to COVID-19. Also, advanced therapies such as deep brain stimulation or levodopa infusion therapy seemed to increase mortality [44]. PD patients possess a higher risk of in-hospital complications due to delirium, adverse drug reactions, syncope, aspiration pneumonia, falls, and fractures [45]. Hence, strategies to prevent these complications are essential.

Most of the PD patients are giving Levodopa/Carbidopa as treatment [37]. To date, there has been no evidence in the association between using these drugs and increased risk of developing COVID-19. Also, there is no report of a specific interaction between dopaminergic medications and antivirals. During the systematic infection, dopaminergic drugs should be continued. However, the use of cough syrup containing dextromethorphan and cyclobenzaprine or nasal decongestants containing pseudoephedrine, phenylephrine, and phenylpropanolamine possible drug interactions which can exacerbate sympathomimetic behaviors should be noted with caution [39,46].

Dopaminergic drugs enhance both motor and nonmotor symptoms of PD, which can capacity to deal with a disease by diminishing off-period duration [46]. A sudden withdrawal of dopaminergic drugs can result in worsening motor symptoms, lethargy, malignant akinesia, etc. [44]. Thus, patients or caregivers should be told that a more abundant supply of oral replacement drugs than usual should be obtained and kept at home.

## 5 MS

MS is a progressive disease of the CNS, primarily immune-mediated, and one of the most common causes of neurological disability in young adults globally. The prevalence is estimated at 2.3 million worldwide [47].

There is no data to indicate a higher risk of COVID-19 in MS patients. However, increased risk of infections such as pneumonia (especially in people with bulbar weakness resulting in aspiration and impaired pulmonary function due to severe quadriparesis) and influenza, across all age groups in MS patients compared with the general population has been demonstrated [48]. Also, hospitalization rates are higher in MS patients with older age, male sex, worse physical disability, and lower socioeconomic status. Intensive care unit (ICU) admission and mortality rates due to infections are also higher [49]. In addition to the increased background risk of infections, MS patients treated with second-generation disease-modifying therapies (DMTs) are exposed to an additional increased risk of infections [50]. When recommending individuals about the dangers of COVID-19 infection, these considerations should be considered.

SARS-CoV-2 is a neurotrophic and neuroinvasive pathogen that can cause demyelination, neurodegeneration, and cellular senescence. The pathogenesis of severe viral infections is associated with systemic inflammatory response syndrome (SIRS) or SIRS-like immune disorders. For SARS-CoV-2 infection, the cytokine storm-induced pro-inflammatory state and chronic viral persistence in oligodendrocytes are responsible for the immune-mediated demyelination [9]. There is a shred of evidence for the association between viral infections and demyelinating diseases. For example, the development of MS is related to Epstein–Barr virus infection [51]. Other common viruses such as varicella-zoster, influenza can cause frequent and severe relapses in patients with MS [52]. If we concentrate on the family of coronavirus, the presence of human CoV in the brain tissue of patients with MS and its RNA has been shown in past epidemics triggered by other coronaviruses. In addition, the anti-CoV antibodies in CSF of MS patients have been detected [53]. Decreased regeneration of myelin occurs during natural aging in mammals and can be intensified after infection with SARS-CoV-2 but postponed until the acute infection is restored. Delayed neurological sequelae resembling MS (ataxia and peripheral neuropathy) can initially be detected post-infection. Differentiation and remyelination oligodendrocyte progenitor cells (OPCs) of affected nerve tissues determine the duration of post-infection symptoms [54]. Because ACE2 is present on OPCs, SARS-CoV-2 infection can negatively impact oligodendrocyte differentiation, leading to chronic or even gradual deterioration of these demyelinating conditions over time [55].

Mechanism of action of DMTs is related to disease pathophysiology, which is the damage to the CNS, myelin sheath, and axonal destruction by T and B lymphocytes, macrophages, antibodies, and complement [56]. Interferon beta and glatiramer acetate, which have an immunomodulating effect, have no impact on the risk of systemic infections [57]. Other agents used in contemporary MS practice have immunosuppressive effects with alterations in lymphocyte number, trafficking, proliferation, and function, with an increased risk of infections, including viral infections and respiratory infections [58]. However, in a case report by Barzegar et al., COVID-19 in a 42-year old MS patient treated with fingolimod, despite comorbidities, was resolved with a good outcome and did not need ICU admission or mechanical ventilation. But, neurologic symptoms were worsening through the infection. Consequently, pseudo exacerbations or relapses should prompt COVID-19 testing during the pandemic without a clear etiology [59].

There are no drugs approved for the treatment of COVID-19, but the FDA has authorized the use of some treatments for those who meet specific criteria. If someone with MS gets COVID-19 and meets the criteria to use an authorized drug, it will be prescribed. Individual risks, including the current use of a DMTs, will need to be considered in this decision-making.

Counseling of patients with MS who wish to consider halting or even stopping MS therapy during the pandemic will be affected by (1) patient factors, such as age and comorbidity, which raise the probability of serious COIVD-19; (2) disease factors, including pre-treatment disease behavior and the preceding 12 months, course of disease and disability; and (3) medication factors, including the change of disease activation [60]. Treatment can be continued during mild viral infections and has to stop in hospitalized with severe or complicated COVID-19. After 4 weeks, or if symptoms have been entirely resolved, treatment can restart.

## 6 Telemedicine and COVID-19

Telemedicine defines as the full spectrum of activities used to provide remote care without actual physical contact with the patient. Telemedicine can help slow the transmission of the virus by decreasing person-to-person contact, especially in patients at higher risk of severe infections (older adults and pre-existing medical conditions) [61]. This plays a crucial role in preventing many patients with neurological diseases from possible COVID-19 exposure. Telehealth can be extremely useful in managing patients with chronic neurological disease, especially those on immunosuppressant drugs. Drug compliance and disease management are critical means of alleviating the severity of infection in these patients [62]. Telemedicine not only eliminates non-essential face-to-face experiences and possible disease transmission during this pandemic but often offers quicker access to specialist care, reduces the cost of patient travel, and is much more convenient for both patients and caregivers [63]. Apparently, the most noticeable weakness of telehealth is the inability to perform a face-to-face physical examination. But, an accurate and detailed neurological examination can be performed through a telehealth platform such as WhatsApp and Skype. These virtual visits allow neurologists to communicate with their patients, continue providing outpatient care, and monitor neurological complications and medication prescriptions while keeping the social distancing [64]. Telemedicine can be an ideal alternative for face-to-face experiences with certain subspecialties, such as epilepsy, where seizure management, medication adjustment, and evaluation and side effects, drug changes, and counseling are the subjects of follow-up visits [63]. Previous experience with SARS indicated that infectious diseases could cause patients to refuse to go to their routine appointments. This can be minimized by promoting telemedicine. Also, rather than suggesting patients and caregivers maintain a stockpile in their homes that may lead to unforeseen consequences, particularly in patients with dementia who are unreliable when taking prescribed medications, physicians can prescribe additional medicines. Therefore, further consideration should be given to introducing and implementing telemedicine [61,62].

## 7 Conclusion

COVID-19 pandemic has disturbed the world and health care system in unprecedented ways. This research examined the recommendations from medical societies about documented evidence of COVID-19 linked to epilepsy, dementia, PD, and MS. The impact of COVID-19 on people with such neurological disorders remains unknown. Clinicians need to share informations, promote research, and offer documented evidence to chronic neurological diseases patients and their families.
